# Gene expression signatures of stepwise progression of Hepatocellular Carcinoma

**DOI:** 10.1371/journal.pone.0296454

**Published:** 2023-12-29

**Authors:** Manisri Porukala, P. K. Vinod

**Affiliations:** Centre for Computational Natural Sciences and Bioinformatics, IIIT, Hyderabad, India; The University of Queensland Faculty of Medicine, AUSTRALIA

## Abstract

The molecular pathogenesis of Hepatocellular Carcinoma (HCC) is a complex process progressing from premalignant stages to cancer in a stepwise manner. Mostly, HCC is detected at advanced stages, leading to high mortality rates. Hence, characterising the molecular underpinnings of HCC from normal to cancer state through precancerous state may help in early detection and improve its prognosis and treatment. In this work, we analysed the transcriptomic profile of tumour and premalignant samples from HCC or chronic liver disease patients, who had undergone either total or partial hepatectomy. The normal samples from patients with metastatic cancer/polycystic liver disease/ cholangiocarcinoma were also included. A gene co-expression network approach was applied to identify hierarchical changes: modules, pathways, and genes related to different trajectories of HCC and patient survival. Our analysis shows that the progression from premalignant conditions to tumour is accompanied by differences in the downregulation of genes associated with HNF4A activity and the immune system and upregulation of cell cycle genes, bringing about variability in patient outcomes. However, an increase in immune and cell cycle activity is observed in premalignant samples. Interestingly, co-expression modules and genes from premalignant stages are associated with survival. THBD, a classical marker for dendritic cells, is a predictor of survival at the premalignant stage. Further, genes linked to microtubules, kinetochores, and centromere are altered in both premalignant and tumour conditions and are associated with survival. Our analysis revealed a three-way molecular axis of liver function, immune pathways, and cell cycle driving HCC pathogenesis.

## Introduction

HCC is the common form of primary liver tumour and the third-most leading cause of cancer-related deaths globally [1, 2]. Major risk factors leading to HCC include viral infections (Hepatitis B—HBV and Hepatitis C—HCV), excessive alcohol and tobacco consumption, exposure to fungal toxins, and Steatotic liver disease (SLD), with 90% of cases arising from the underlying chronic liver disease [3]. While HBV-driven HCC is prevalent in East Asia and Africa, HCV infections are most common in the US and Europe. SLD is emerging as the leading risk factor of HCC, especially in the West, owing to the rise in obesity and diabetes [4]. Despite continuous advances and management strategies designed to mitigate the incidence of HCC, its mortality rates have been rising over the last two decades. The major caveat in reducing the incidence of HCC is the detection at an early stage since more than 50% of HCC cases are diagnosed at advanced stages [5]. Therefore, a better understanding of HCC pathogenesis and its molecular underpinnings will help reduce the rising cases.

Most HCC cases develop in the background of unresolved chronic inflammation [6] that triggers a persistent healing response [7]. The unbalanced healing response disturbs the architecture of the liver, leading to fibrosis, followed by cirrhosis. The regenerating nodules produced during cirrhosis fuel the transformation of hepatocytes to premalignant lesions called dysplastic nodules. These premalignant lesions develop into early HCC (eHCC) and progressive HCC (pHCC). Although this stepwise progression from chronic liver disease to tumour state is widely prevalent in HCC, about 20% of cases arise from a non-cirrhotic background [8]. While most non-cirrhotic HCCs develop from metabolic syndrome [9], HBV or HCV infection can also lead to HCC from accelerated fibrosis without cirrhosis [8, 10]. Hence, it is crucial to consider the existence of multiple trajectories to HCC when developing diagnostic markers.

Due to the inherently complex nature of HCC development, managing patients is also quite challenging. Surgical resection is the primary treatment for HCC patients with preserved liver function but is prone to recurrence in about 70% of the cases within a few years [5]. Liver transplantation is another option for patients not eligible for resection but is limited by the availability of donors [11]. Under the circumstances where resection or liver transplantation is not amenable, liver-directed medication fails, or recurrence is seen post-resection, systemic therapy is chosen [5]. Systemic therapy in the form of tyrosine kinase inhibitors and immunotherapy targeting immune checkpoints have been developed for treating advanced-stage HCC [12]. Further, treatment strategies must also consider underlying liver disease along with tumour stage [13, 14], which may account for differences in the risk of HCC recurrence among patients [15].

The advancement in the high throughput techniques (next-generation sequencing) is helping to map the molecular changes of HCC at genomic, transcriptomic, and epigenetic levels [6, 16]. This information provides insights into the various signalling pathways involved in hepatocarcinogenesis. These include differentiation and development pathways (Wnt/β-catenin, Notch Hedgehog signalling), genomic stability and cell cycle (TP53, RB1), telomerase (TERT), growth and cell proliferation (PI3K/AKT/mTOR, RAS/MAPK, EGF/EGFR), angiogenesis (VEGF/VEGFR, PDGF/PDFGR) and chromatin remodelling (ARID1A/ARID1B/ARID2 and MLL signalling) [6, 17]. However, the current understanding of the interplay of various signalling pathways in HCC is far from complete.

Molecular profiling distinguishes diverse subgroups of HCC that are otherwise indistinguishable by conventional histological methods [18]. Gene expression changes in tumour samples are used to predict recurrence and stratify patients into high and low-risk groups [19]. In liver cancer, genes that show a fold change in expression between the normal and tumour samples are a better predictor of survival than considering candidates based on tumour samples alone [20]. Gene expression profile of tumour-adjacent normal tissue is also reported to predict HCC recurrence [21]. Prediction models proposed in these studies are based on differentially expressed genes in tumours or pre-defined gene signatures.

A recent study on a comprehensive analysis of tumour samples, tumour-adjacent normal samples, and normal healthy samples showed that tumour-adjacent normal samples represent an intermediate transcriptomic state between the other two [22]. Therefore, there is a need to explore the multi-step progression of HCC through different trajectories to gain further insights into the molecular mechanisms and develop predictive models. Network-based approaches provide a suitable platform to extract meaningful information from omics data, hypothesis generation, stratification of disease classes, and discovery of biomarkers [23]. In the present work, we aim to understand the molecular pathogenesis of HCC sequentially from normal to tumour through different premalignant stages. A network-level analysis of the transcriptomic profile of tumour samples and tumour-adjacent normal samples in different liver damage conditions was performed to obtain insights into the transition from normal to precancerous to cancer state. The hierarchical changes: modules, pathways, and genes related to HCC progression and survival prediction were identified.

## Methods

### Dataset description

**Fig 1:**
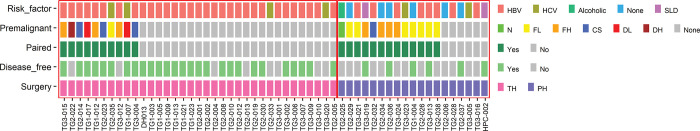
Clinicopathological features of tumour samples in the Korean cohort. This includes the surgery type (TH/PH), disease recurrence, whether the tumour sample has an adjacent normal sample, the premalignant stage of the tumour-adjacent normal, and the risk factor (HBV, HCV, Alcoholic, None, SLD).

Bulk RNA-seq transcriptomics data of HCC progression was obtained from GEO with accession number GSE148355. The dataset consists of tumour and non-tumour samples from HCC patients or patients with the chronic liver disease treated with either total hepatectomy (TH) or partial hepatectomy (PH) at Seoul National University Hospital. Clinical information is available for 54 tumour samples (35 are from TH patients and 19 from PH patients). The dataset also comprises 47 premalignant and 15 normal samples. The normal samples were from patients with metastatic cancer/polycystic liver disease/or cholangiocarcinoma. All non-tumour liver tissues have no evidence of hepatic fibrosis or viral hepatitis. Out of these 47 premalignant samples, 24 are tumour-adjacent normal samples. The premalignant stages include Fibrosis Low (FL)– 10 samples, Fibrosis High (FH) -10 samples, Cirrhosis (CS)– 10, Dysplastic nodule low grade (DL)– 10 samples, and Dysplastic nodule high grade (DH)– 7 samples. All samples were collected after receiving written informed consent from the patients, and the original study was approved by the Institutional Review Board of Seoul National University Hospital. This dataset is referred to as the Korean cohort. The plot summarising clinicopathological features of tumour samples is given in Fig 1.

In addition, we used HCC datasets from TCGA (TCGA-LIHC) and GEO (GSE14520) with available clinical information. TCGA gene expression data and clinical data were obtained from UCSC Xena (https://xena.ucsc.edu/). TCGA-LIHC comprises 316 tumour samples with clinical information, and 39 of them have paired normal samples. GSE14520 is a microarray based (GPL3921 platform) gene expression profiling from HCC patients treated with surgical resection. The dataset includes gene expression data of 210 tumour and 210 adjacent normal samples with associated clinical information and is referred to as the Chinese cohort.

### Network-based approach

**Fig 2:**
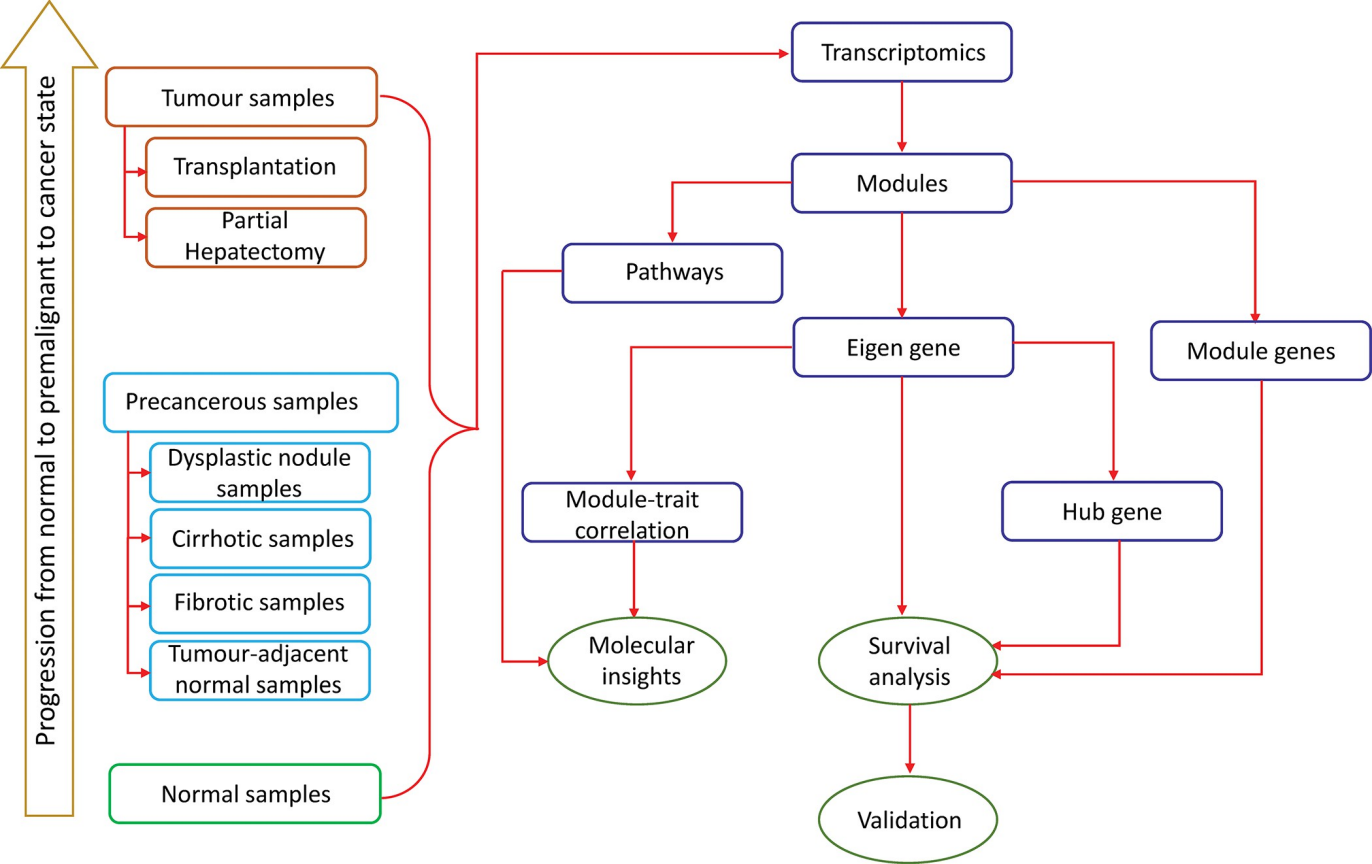
The workflow to study the progression from normal to precancerous to cancer state in HCC.

A systems-level analysis was designed to study the pathogenesis of HCC at multiple levels: modules, pathways, and genes **(Fig 2)**. The analysis pipeline was applied to three groups of samples: (a) only tumour samples, (b) adjacent normal and tumour samples, and (c) all normal and premalignant samples. To identify modules, the co-expression network was constructed from gene expression data of the Korean cohort using weighted gene co-expression network analysis (WGCNA) in R [24, 25]. FPKM values were transformed to log_2_(FPKM + 1), and the top varying genes were selected using the rowVars function to construct the correlation (Pearson) matrix s_ij_ for WGCNA. A signed network was built by transforming the correlation matrix to an adjacency matrix (a_ij_) using the power adjacency function and soft thresholding (a_ij_ = s^β^_ij_). Scale-free topology criteria was used to choose the power β. Subsequently, a Topological Overlap Matrix (TOM) was computed from the adjacency matrix, followed by dendrogram construction using 1 –TOM. Modules were identified from the dendrogram using the dynamic cut tree algorithm, and module eigengene expression (ME) for each module was calculated using singular value decomposition (SVD).

Modules significantly correlating with disease-free survival (DFS) and other clinical traits were identified. Categorical traits such as surgery/treatment (TH and PH) and premalignant state were converted into continuous numerical values to compute correlation with different modules. For surgery, PH and TH were binarized as 1 and 2, respectively. The premalignant states were converted to numerical with 1, 2, 3, 4, 5, and 6 indicating normal, FL, FH, CS, DL, and DH, respectively. Hub genes from modules were extracted based on module membership (MM > 0.8) and intramodular connectivity.

Candidate genes were selected for univariate survival analysis based on the modules that correlated with DFS under each condition. Samples were dichotomised into two groups based on the median gene expression profile of candidate genes, and survival analysis was performed using the survival R package [26]. Further, module preservation analysis [27] was carried out using TCGA data as the test set to access the biological relevance of modules identified from the Korean cohort. The Z_summary_ statistics was used to evaluate whether the module is preserved between the reference set (Korean cohort) and test set (TCGA) with the following cut-off:

$$module\;preservation(Z_{summary})=\left\{ \begin{array}{l} moderate,\;{2<Z}_{summary}<10\\ \;strong,\;Z_{summary}\geq 10 \end{array}\right.$$

### Pathway enrichment analysis

The enrichment analysis of module genes was performed using Enrichr [28] to identify dysregulated pathways. The ClueGO Cyctoscape plugin was used with default settings to visualise the interrelations of the GO biological terms associated with modules [29].

## Results

### Co-expressed modules of tumour samples

**Fig 3:**
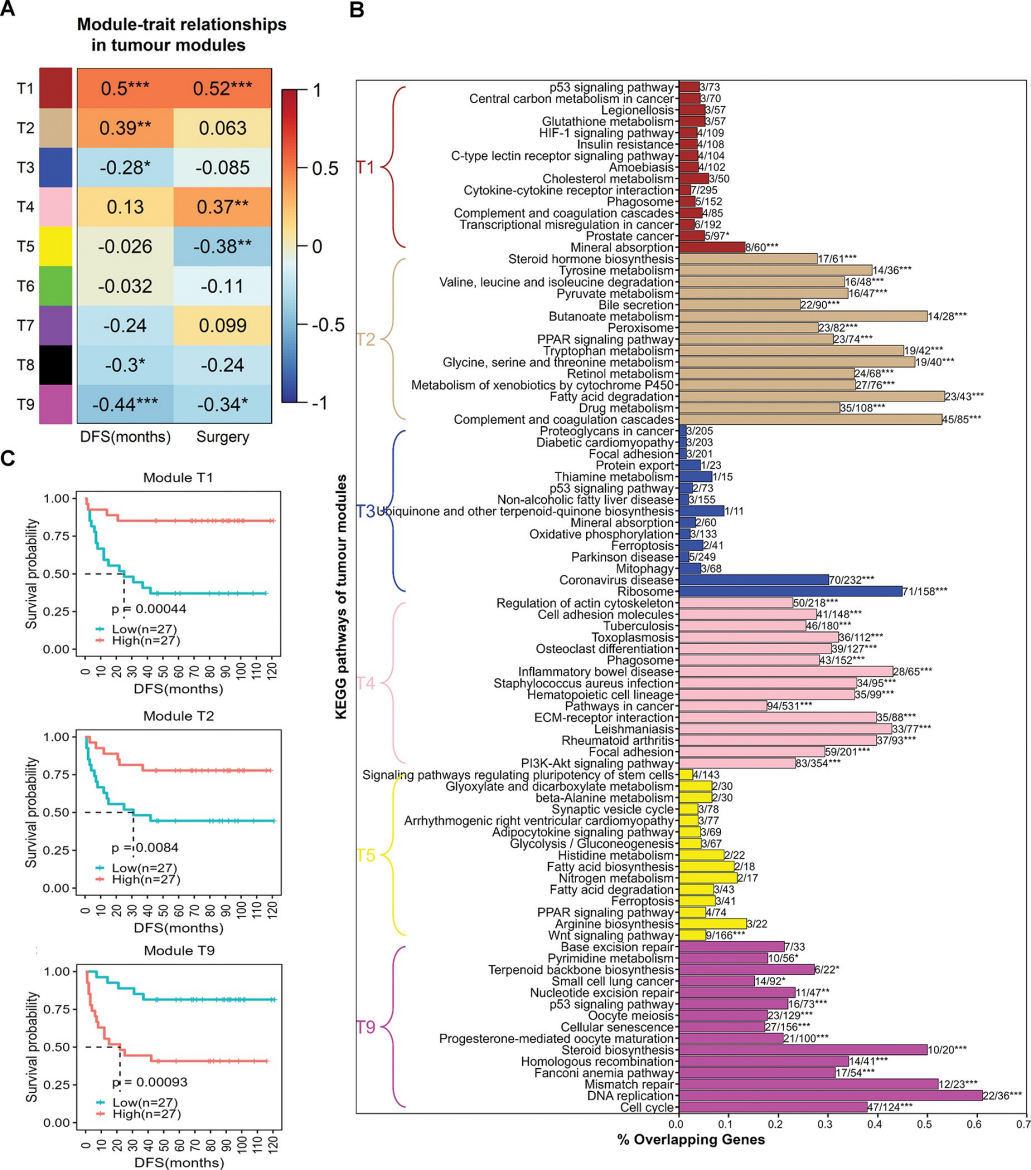
Co-expressed modules of HCC. **(A)** Module-trait correlations of tumour samples. DFS representing disease-free survival is a continuous variable. Surgery (treatment) is a binary variable with partial hepatectomy (PH) represented as 1 and total hepatectomy (TH) as 2. *** indicates p-value < 0.001, ** indicates 0.001 ≤ p-value < 0.01, * indicates 0.01 ≤ p-value < 0.05. **(B)** KEGG pathway enrichment of tumour modules. For each module, 15 most significant pathways sorted according to adjusted p-value are displayed (bottom to top within each module). *** indicates adjusted p-value < 0.001, ** indicates 0.001 ≤ adjusted p-value < 0.01, * indicates 0.01 ≤ adjusted p-value < 0.05. The number of overlapping genes and the total number of pathway genes are shown to the right of each bar. **(C)** Survival analysis based on eigengene expression of tumour modules. Samples are classified into high and low-expression groups based on the median of eigengene expression of each module. ‘p’ indicates the p-value of survival analysis.

The co-expression pattern of genes within tumour samples (Korean cohort) was studied using top-varying genes. We found five modules (T1, T2, T3, T8, and T9) that significantly correlated with DFS **(Fig 3A)**. Coincidentally, T1 and T9 modules are significantly correlated with the surgery/treatment (i.e., PH or TH) as well. An increase in DFS is associated with TH as the treatment. This is in agreement with the original study [30], which shows TH group has better DFS compared to patients undergoing PH treatment, although there were no differences in grade/stage of tumour in these two groups.

KEGG pathway enrichment of the T9 module showed that cell cycle-related pathways play an important role in governing the survival of a patient post-treatment **(Fig 3B)**. The eigengene expression pattern of this module shows that DFS decreases with an increase in cell cycle activity. The T1 module includes cancer-related genes and pathways relevant for DFS prediction post-treatment. The T2 module that is positively correlated with DFS is enriched for amino acid metabolism, fatty acid degradation, and xenobiotic metabolism, indicating the capability of the liver to carry out its basic functions post-treatment, thus improving survival. It is also associated with complement and coagulation cascades. The T3 module is negatively correlated with DFS and is associated with ribosomes. T4 and T5 modules are associated with the treatment and are related to ECM and regulation of the Wnt signalling pathway, respectively.

Since some modules showed a significant correlation with DFS, we checked if their respective eigengene expression could be used to identify differences in survival probability **(Fig 3C)**. We observed that the corresponding eigengene expression of DFS modules (median) also performs well in predicting the survival probability. Further, the hub genes of these modules also predicted the differences in survival probability and helped us to identify biomarkers. **S1 Dataset** shows the list of hub genes in each module and their association with the DFS of patients. The low expression of the macrophage scavenger receptor gene MARCO is associated with poor DFS in HCC patients. The evaluation of MACRO protein expression by immunostaining in HCC shows that its level decreases as the disease condition worsens [31]. CELC1B is a platelet-related gene, and its expression is related to immune cell infiltration [32]. CFP regulates the complement pathway, and its expression correlates with the infiltration of immune cells [33]. Genes related to the lectin pathway of complement activation (COLEC10, FCN2, FCN3) are also DFS hub genes of the T1 module. The expression of CRHBP, which mediates the reaction between the corticotropin-releasing hormone and its receptor, is also a predictor of DFS in HCC. The hub genes of the T2 module are related to metabolic processes, including TAT, a tumour suppressor gene. MTHDF1, involved in the interconversion of 1-carbon derivatives of THF, is also a DFS hub gene. Hub genes associated with microtubules and chromosomes from the T9 module are also good predictors of DFS in HCC. The modules identified from tumour samples of the Korean cohort are also preserved in tumour samples of TCGA **(Figure S1 in S1 Text)**. Further, the genes from the above modules also show significant survival differences in TCGA tumour samples **(Table S1 in S1 Text)**.

### Progression from precancerous to cancerous state

Module-trait correlation with tumour samples revealed that modules significantly correlated with DFS also captured the differences in surgery a patient has undergone. Based on these observations, we hypothesised that there could be differences in the mechanism of precancerous to cancerous progression between two groups of patients undergoing either TH or PH. Therefore, we investigated the differences in the progression by identifying co-expression modules in each group from both tumour and tumour-adjacent normal samples and correlating them with disease conditions.

**Fig 4:**
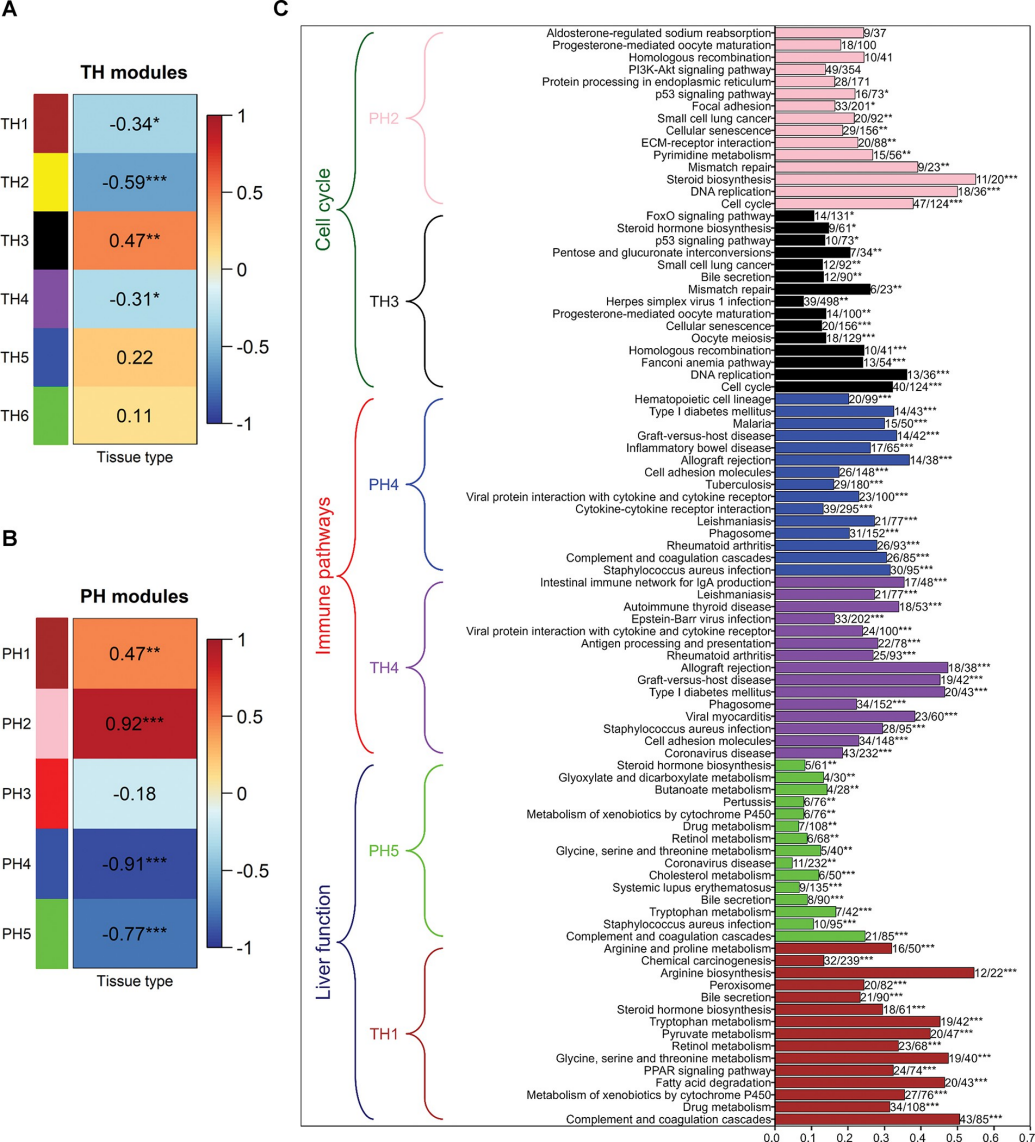
Progression from precancerous to cancer state. Module-trait correlations in **(A)** TH and **(B)** PH treatment groups. Tissue type is a binary variable with the precancerous state as 1 and cancer state as 2. *** indicates p-value < 0.001, ** indicates 0.001 ≤ p-value < 0.01, * indicates 0.01 ≤ p-value < 0.05. **(C)** KEGG pathway enrichment of precancerous to cancer modules. For each module, the 15 most significant pathways sorted according to adjusted p-value are displayed (bottom to top within each module). *** indicates adjusted p-value < 0.001, ** indicates 0.001 ≤ adjusted p-value < 0.01, * indicates 0.01 ≤ adjusted p-value < 0.05. The number of overlapping genes and the total number of pathway genes are shown to the right of each bar.

The progression from precancerous to cancerous state in both groups shows that liver function is affected in tumour samples. Tumour samples show a decrease in liver function (TH1 module in TH group, PH5 module in PH group) and compromised immune-related pathways (TH4 module in TH group, PH4 module in PH group) **(Fig 4)**. The transcription factor enrichment of TH1 and PH5 modules based on ENCODE data shows HNF4A as an associated transcription factor. Modules capturing cell cycle changes in both groups (TH3 module in TH, PH2 module in PH) show a positive correlation in tumour samples. We observed significant correlations to these biological processes in PH compared to TH (**Fig 4A and 4B)**. PH4 module shows a higher negative correlation compared to the TH4 module with respect to tumour samples, suggesting that immune response genes are significantly downregulated in PH compared to TH. Another feature difference in precancerous to cancer progression is that the cell cycle module shows a very high positive correlation in PH samples (PH2 module) compared to TH samples (TH3 module). A comparison of these modules in both groups shows some overlap, but the majority of genes are unique to a particular module in a group **(Figure S2A in S1 Text)**. These sets of unique genes may account for the difference in the precancerous to cancer progression. This analysis gives a global picture of precancerous to cancer progression in both TH and PH groups fuelled by deviations in liver function, cell cycle, and immune response **(Fig 4)**.

In addition to these observations, DEGs comparing tumour versus adjacent normal in both the groups also supports this stark difference in cell cycle and immune response genes between the two groups **(Figures S2B and S3 in S1 Text)**. Further, we also observed that oxidative phosphorylation genes are downregulated in the TH group, while genes of choline metabolism in cancer and arginine biosynthesis are upregulated in PH. Genes of Th1 and Th2 cell differentiation, Th17 differentiation, and complement and coagulation cascades are downregulated in the PH group. We also performed module preservation analysis using TCGA samples **(Figure S4 in S1 Text)**. The modules from the PH and TH groups show medium to high preservation in TCGA samples.

### Co-expressed modules of normal and premalignant samples

**Fig 5:**
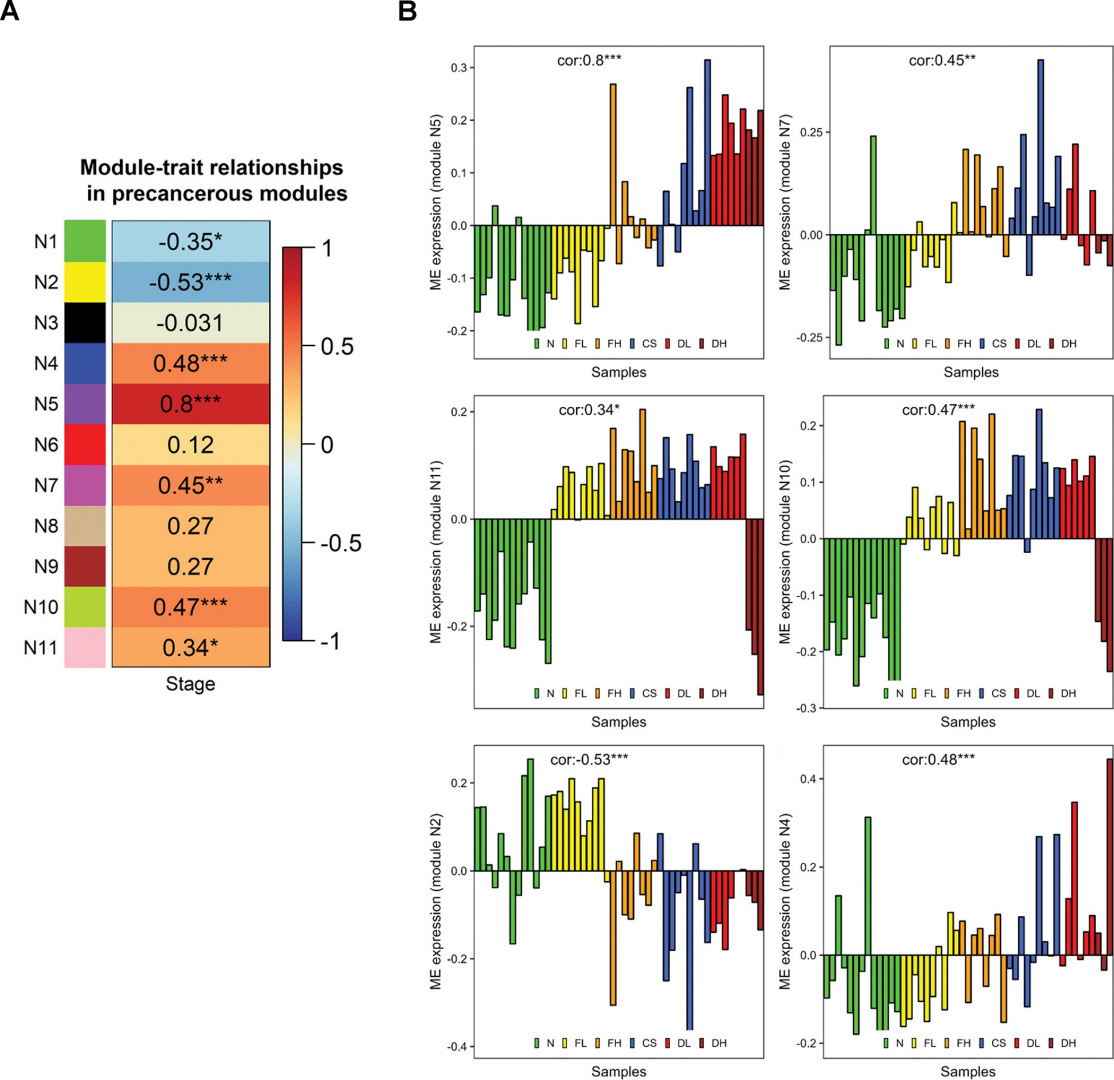
Co-expression modules of normal and premalignant conditions. **(A)** Module-trait correlations of premalignant samples. The stage represents different premalignant conditions. **(B)** Eigengene plots for individual premalignant modules showing correlation with premalignant stage. *** indicates p-value < 0.001, ** indicates 0.001 ≤ p-value < 0.01, * indicates 0.01 ≤ p-value < 0.05.

The premalignant condition (47 samples) in the dataset ranges from fibrosis (low and high grade) to cirrhosis and dysplastic nodule (low and high). We also included 15 normal samples to capture the changes from normal to premalignant lesions sequentially based on co-expression analysis. We found seven modules significantly correlating with premalignant stages **(Fig 5A)**. The N5 module showed a significantly high correlation to premalignant stages with low expression in normal and FL stages and high expression in FH, CS, DL, and DH stages **(Fig 5B)**. This module is associated with cellular response to type 1 interferon, cytokine-mediated signalling, and defense response to the virus **(Table S2 in S1 Text)**. The N7 module is related to neutrophil-mediated immunity and inflammatory response. The N10 module is also positively correlated to premalignant stages, capturing the changes in gene expression that occurred early in the FL stage. These early changes are associated with the complement coagulation cascade, ribosome machinery, and lipid metabolic process. The N11 module showed a gene expression pattern similar to the N10 module and is associated with mitochondrial oxidative phosphorylation.

The N2 module is negatively correlated with the premalignant stage and is enriched for metabolic pathways linked to liver function and HNF4 transcriptional activity. The eigengene expression shows that liver function is compromised in the late premalignant stages **(Fig 5B)**. Intriguingly, the N4 module that is positively correlated with premalignant stages is associated with cell cycle pathways, showing the onset of the tumourigenesis process.

A previous study on HCC showed that hepatic injury and regeneration (HIR) signature (233 genes) is a good predictor of DFS using premalignant samples from the Chinese cohort [34]. We verified the overlap of the HIR signature with premalignant modules identified through our analysis. We observed that only the N3 module, which is not associated with premalignant states, showed a significant overlap of 61 genes with the HIR signature **(Figure S5 in S1 Text)**. The N3 module is associated with immune pathways and cellular senescence.

**Fig 6:**
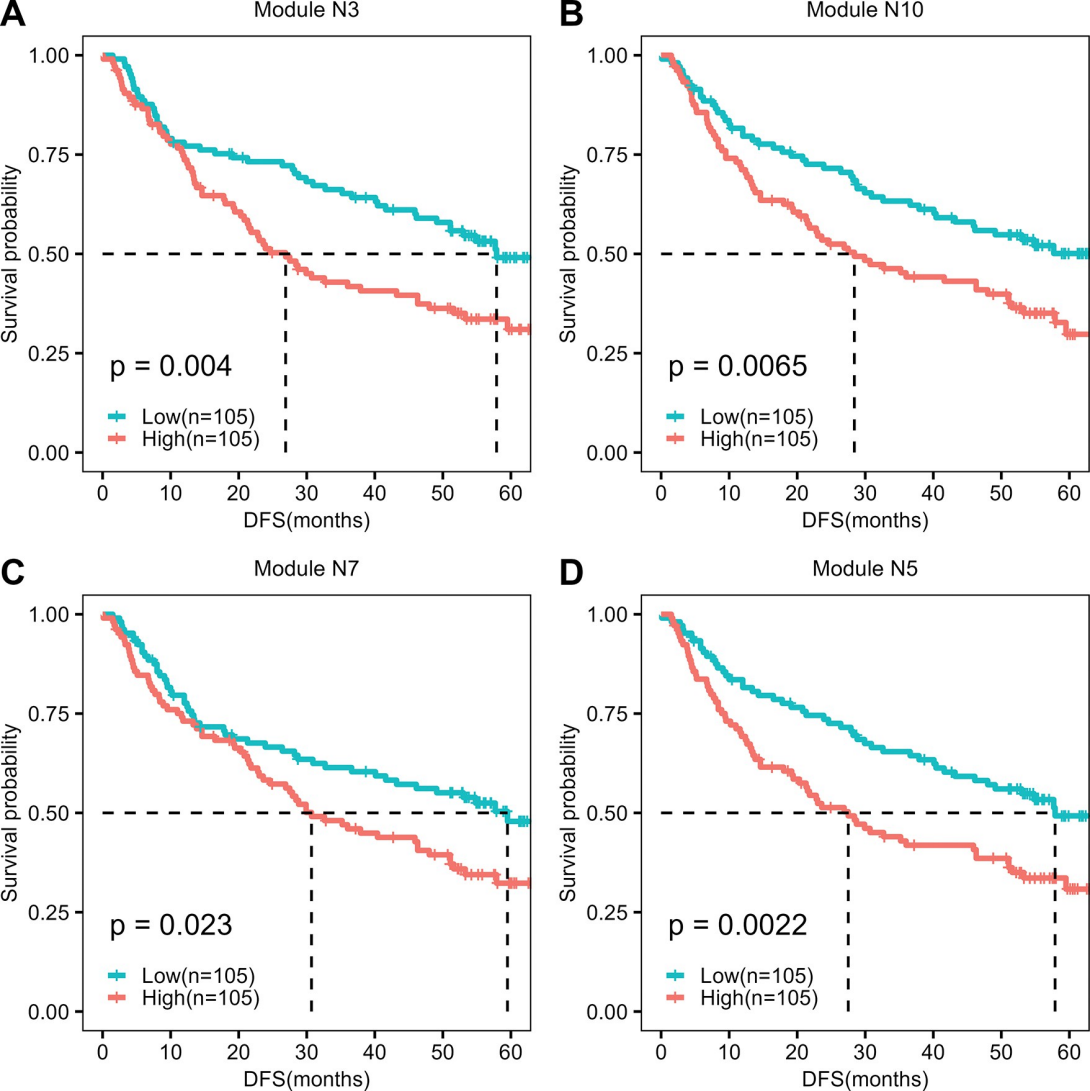
Eigengene-based survival analysis using tumour-adjacent normal samples in the Chinese cohort. The eigengene expression of each premalignant module (obtained from the Korean cohort) was calculated using the tumour-adjacent normal samples in the Chinese cohort. Samples are classified into high and low-expression groups based on the median of eigengene expression of each module. ‘p’ indicates the p-value of survival analysis.

We also tested the ability of individual modules to predict the DFS. For this purpose, the Chinese cohort was chosen due to the large sample size with clinical information compared to the Korean cohort. For each module (N1 –N11) identified in the Korean cohort, we calculated the corresponding eigengene from paired normal samples of the Chinese cohort, followed by survival analysis based on eigengene expression. The eigengene expression of the N3 module predicts DFS (p-value = 0.004) with a high expression value associated with poor survival **(Fig 6)**. It was observed that 29 out of the 61 intersecting genes between the N3 module and the HIR signature performed well in predicting the DFS in univariate Cox regression analysis **(S1 Dataset)**. These include genes, PLK2, ODC1, WWC1, MYC, DDX21, SOCS3. The high expression of these genes is associated with poor survival. 13 genes out of 96 non-intersecting genes also showed good predictability of DFS **(S1 Dataset)**. Interestingly, we also observed that eigengene expression of modules associated with premalignant stages (N10, N7 and N5) predicted DFS based on normal/premalignant samples **(Fig 6).** The N5 module yielded the best p-value of 0.0022 in the DFS analysis. THBD (p-value = 0.00035) and BCL2L1 (p-value = 0.0007) are top candidate DFS genes from the N7 module **(Table S3 in S1 Text)**. THBD is a classical marker for dendritic cells (DCs). Increased DCs are associated with early relapse of HCC [35]. BCL2L1 promotes invasion and inhibits apoptosis of liver cancer cells [36]. High expression of FOS (p-value = 0.005) and JUN (p-value = 0.0015) in the N5 module are also associated with poor DFS **(Table S3 in S1 Text)**. Thus, by extracting modules of co-expressed genes from premalignant samples, we identified biomarkers for DFS prediction.

### Cell cycle-related pathways change in progression from normal to precancer to HCC

**Fig 7:**
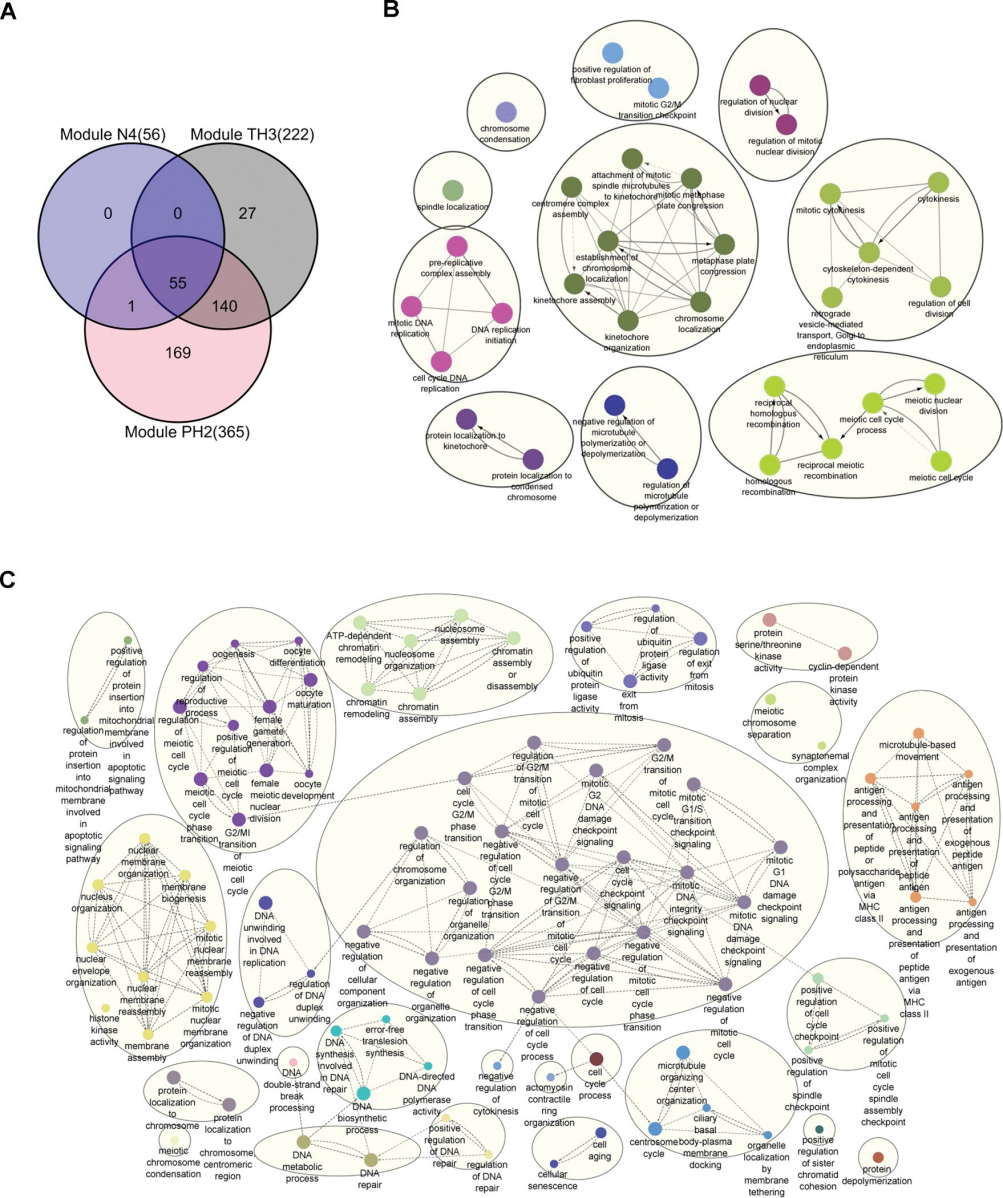
Cell cycle alterations from normal to HCC transition. **(A**) Venn diagram showing the overlap of cell cycle genes with modules significantly enriched for cell cycle-related pathways in premalignant and malignant samples (PH and TH groups). **(B)** Network of GO biological processes of cell cycle genes in premalignant module N4. **(C)** Network of GO biological processes of cell cycle genes in precancerous-cancer module TH3.

It was observed that cell cycle-related pathways were enriched in premalignant samples (N4 module). Similarly, TH3 and PH2 modules from the precancerous to the cancer stage of TH and PH samples were also associated with the cell cycle. The overlap of these module genes with the cell cycle-related genes obtained from the GO term **(S1 Dataset)** showed that 55 genes are common and found in precancerous stages **(Fig 7A)**. We observed an increase in cell cycle-related genes with progression from precancer to cancer in TH3 and PH2 modules, having 222 and 365 genes, respectively. There are 169 cell cycle genes unique to the PH2 module.

**Fig 8:**
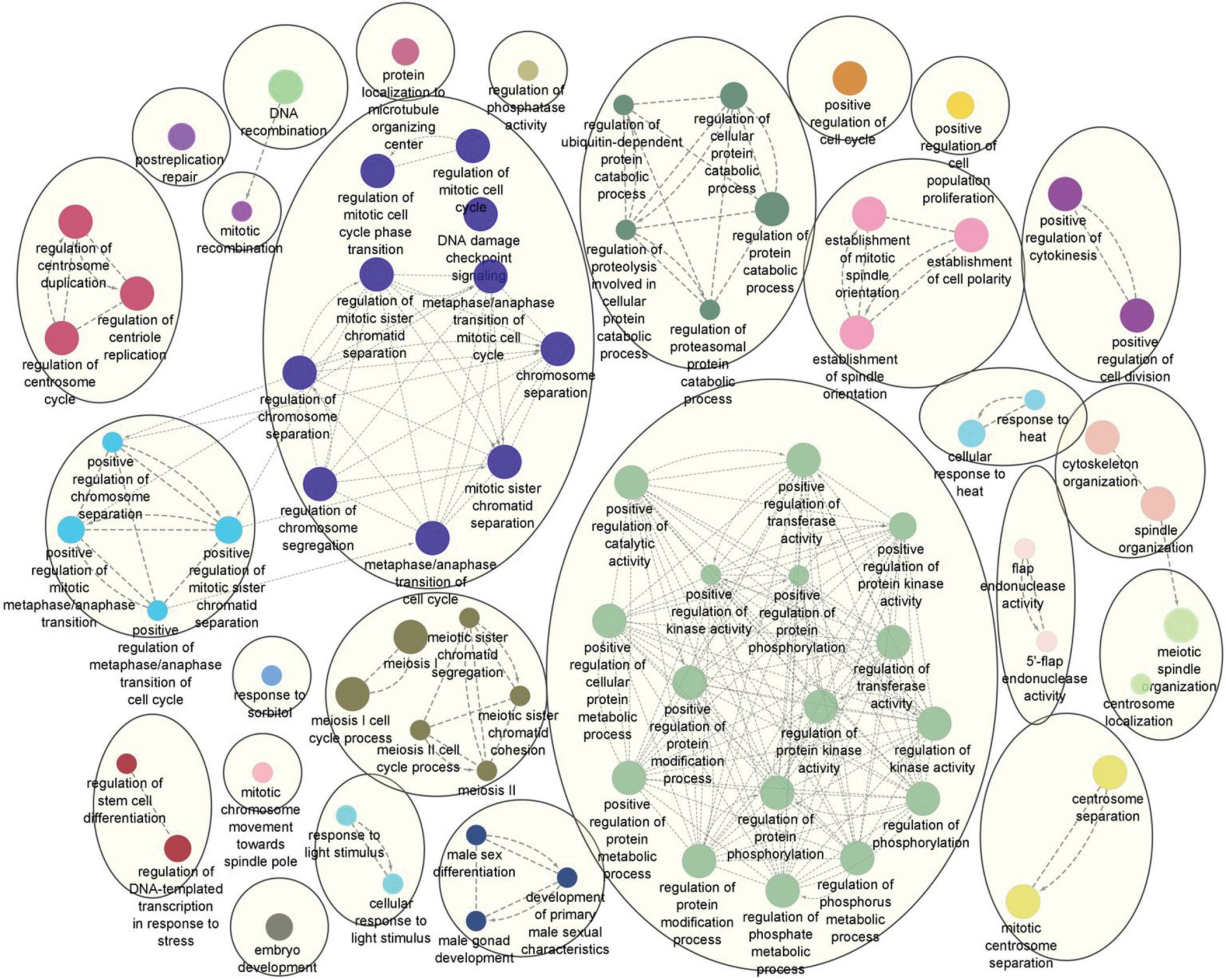
Network of GO biological processes of cell cycle genes in precancerous-cancer module PH2.

To gain further insights into the cell cycle processes, genes of the individual modules (N4, TH3, PH2) overlapping with cell cycle genes (56, 222, 365 genes, respectively) were visualised using GO biological processes with ClueGO Cytoscape plugin. The 55 common genes map to biological processes related to the kinetochore, microtubule and chromosome **(Fig 7B).** GO terms unique to TH3 and PH2 modules suggest the progression differences from precancerous to cancer state between TH and PH conditions. Checkpoint signalling, negative regulation of the cell cycle process, DNA repair process, and regulation of exit from mitosis are observed in the TH3 module but not in the PH2 module **(Figs 7C and 8)**. On the other hand, the PH2 module shows positive regulation of cell cycle, proliferation, cell division, and cytokinesis, along with positive regulation of protein metabolic processes. There is an increase in the number of genes related to microtubule spindle organization compared to N4 and TH3 modules. Further, DFS cell cycle genes related to microtubules, kinetochores, and centromere also overlap with genes of the N4 module, suggesting some of these changes are associated with premalignant stages.

## Discussion

Understanding the molecular mechanisms involved in the progression of HCC through multiple trajectories is crucial for improved diagnosis, prognosis, and treatment. In this direction, we investigated publicly available transcriptomics data of HCC patients undergoing liver transplantation or resection treatment. Gene co-expression network-based framework was employed to get molecular insights from the transcriptomics data of tumour samples, tumour- adjacent normal samples in different premalignant states, and normal samples. This approach identified modules of co-expressed genes, pathways, and genes that characterize different trajectories and predict DFS based on premalignant and tumour samples.

Modules and genes related to the cell cycle, immune system, ribosome, and liver metabolic pathways were good predictors for DFS using tumour samples **(Fig 3 and Table S1 in S1 Text)**. An increase in the ribosome and cell cycle activity and a decrease in the expression of immune (complement system) and liver metabolic genes are associated with poor DFS. Liver function and proliferation are shown to be mutually exclusive, and the transition to proliferation occurs with the inhibition of liver function [37]. HCC occurrence and progression are related to the interaction between viruses and ribosomes [38]. A decrease in the complement system also indicates a change in the immune infiltration patterns. DFS modules were also associated with the treatment (surgery) given to patients: PH and TH **(Fig 3A)**. In addition, we also found a tumour module (T4) linked to ECM to be associated with treatment.

The network analysis of patients who have undergone PH and TH was performed independently, including the tumour and corresponding tumour-adjacent normal samples, to understand the differences in progression. We observed that the same biological processes are affected to a different extent in TH and PH groups. Both groups show a decrease in liver function and immune system and an increase in cell cycle activity. However, the tumour samples in the PH group show a very high correlation to these biological processes **(Fig 4)**. This indicates that the extent of immune suppression and decrease in liver function is related to cell cycle activity in tumour samples, bringing about the variability in the outcomes. This view contrasts with our observations from modules identified from normal and premalignant samples. We observed an increase in immune activity and cell cycle gene expression and a decrease in liver function. An increase in immune activity may be associated with the antiviral mechanism (most patients have HBV infection) by interferon signalling. Genes of immune modules (N5, N7, N10) show some overlap with downregulated immune modules (TH4 and PH4) specific to tumour samples. FOS and JUN are part of the upregulated module in premalignant samples and downregulated modules in tumour samples. This suggests a shift in immune activity from a premalignant state to tumour state. Pro-inflammatory M1 marker CCL2 decreases in tumour modules TH4 and PH4 but increases in the premalignant state. The expression of fibrotic genes EGR1, JUND, KLF2 and TAGLN also decreases in tumour samples (PH4 module).

On the other hand, genes related to liver function decrease in premalignant and tumour samples. HNF4A, which controls liver function, is known to be inhibited by increased inflammation (immune activity) in liver fibrosis [39, 40]. The expression of HNF4A leads to the restoration of metabolic function and reversing (attenuation) of liver fibrosis and cirrhosis via controlling macrophages and hepatic stellate cells [41]. HNF4 drives the transition of macrophages to the M2 phenotype. We hypothesise that the mutual antagonism between HNF4A and immune activity plays a role in HCC progression. An increase in inflammation may result in the inhibition of HNF4A with an increase in cell cycle activity. A progressive loss of HNF4A activity is observed in liver diseases (SLD) compared to HCC [42]. We observed that the transition from normal to premalignant to tumour state is also characterised by an increase in cell cycle activity. Genes related to mitotic spindle organisation are present in the premalignant state, and some of them are also DFS genes in tumour samples. An increase in the expression of genes involved in the maintenance of genomic integrity is associated with chromosomal instability (CIN), which is a prognostic factor in multiple cancers [43, 44]. There is also an emerging link between CIN and tumour immunity.

We observed that multiple (N3, N7, N5, N10) modules from premalignant samples are good predictors of DFS **(Fig 6)**. The N3 module showed some overlap with the HIR signature, which was earlier proposed for DFS prediction. However, we identified three more modules that can be used for the prognostic task. These modules are associated with the immune system. We obtained the best performance (p value = 0.0022) with the eigengene expression of the N5 module **(Fig 6D)**. These modules are associated with premalignant conditions (fibrosis, cirrhosis), and an increase in the eigengene expression is associated with poor survival. This suggests that early relapse can also be predicted based on tumour-adjacent normal immune environment. Most studies on HCC relapse are based on immune cell recruitment in tumour samples. Early-relapse HCC cases have increased recruitment of dendritic cells (DC) and CD8^+^ T cells compared with primary tumours [30, 35]. However, our study showed that the gene expression of tumour-adjacent normal samples of HCC patients contains multiple signatures relevant to predicting DFS.

## Limitations

This study on HCC progression was performed solely based on the transcriptomics data. A comprehensive view of progression should also account for alterations at the whole genome (somatic mutations, copy number variations) or epigenome (histone modifications, methylation) level that may drive transcriptional changes. Further, proteomic profiling during the stepwise progression of HCC is required to confirm the changes at the transcriptomic level. The current analysis is performed on the HCC cohort, which is mostly driven by HBV infection. Given the increasing incidence of SLD-driven HCC, it would be relevant to study the SLD cohort using a similar strategy. Further validation of the work is required to study the transition from different premalignant conditions using larger-size cohorts.

## Conclusion

Overall, the network-level analysis of gene expression of HCC in different scenarios, including only tumour samples, tumour and tumour-adjacent normal samples, and normal and premalignant samples, showed that pathways relating to liver function, cell cycle, and immune system are at the interplay of the molecular pathogenesis of HCC. The analysis of precancerous to cancer transition revealed that similar pathways are affected to different extents in PH and TH treatment groups. The characterisation of cell cycle changes showed that the TH group is associated with the negative regulation of cell cycle in contrast to positive regulation of the cell cycle in the PH group. Further, we showed that the gene expression profile of premalignant conditions serves as early biomarkers of HCC and its recurrence. We conclude that this may be due to dynamic changes in gene expression of the biological processes during the stepwise progression of HCC.

## Supporting information

S1 Text Supplementary figures and tables. (PDF)

S1 Dataset List of genes. Survival genes based on modules from tumour samples, and normal and premalignant samples; List of genes in each module; HIR signature; Cell cycle genes.
(XLSX)
