# Supplementary material for: Gene expression signatures of stepwise progression of Hepatocellular Carcinoma
Source: PLoS One. 2023 Dec 29;18(12):e0296454. doi: 10.1371/journal.pone.0296454 (PMC10756545; doi:10.1371/journal.pone.0296454)
Supplement: S1 Text — (PDF) [file pone.0296454.s001.pdf]

Supplementary Information

Gene expression signatures of stepwise progression of Hepatocellular Carcinoma

Manisri Porukala and P K Vinod*

Centre for Computational Natural Sciences and Bioinformatics, IIIT, Hyderabad-500032, India

*Correspondence: vinod.pk@iiit.ac.in

Phone: +91 40-66531177

Table S1: Survival analysis of genes from modules T1, T2 and T9 on tumour samples of the Korean cohort and their validation on TCGA tumour samples. Genes with p-value <= 0.01 are shown.

	Gene	Korean		TCGA	
		**p-value**	**Hazard ratio**	**p-value**	**Hazard ratio**
**Module T1**	COLEC10	4.4e-04	0.18	4.2e-03	0.62
	CRHBP	4.4e-04	0.18	4.1e-04	0.55
	CFP	6.0e-04	0.18	3.3e-04	0.55
	CETP	1.8e-03	0.23	1.7e-03	0.59
	CD5L	1.8e-03	0.23	2.4e-03	0.60
	TIMD4	2.5e-03	0.24	1.1e-03	0.58
	FCN3	3.3e-03	0.25	5.0e-03	0.62
	ANKRD55	3.5e-03	0.25	1.2e-03	0.58
	DNASE1L3	3.6e-03	0.25	5.0e-05	0.51
**Module T2**	LPA	7.0e-04	0.21	4.4e-04	0.56
	ABCA9	1.4e-03	0.22	1.8e-04	0.53
	CFHR4	5.3e-03	0.28	3.4e-03	0.61
	HPX	7.4e-03	0.29	8.3e-03	0.64
	BDH1	7.7e-03	0.30	4.2e-03	0.62
	ASGR2	9.4e-03	0.30	4.8e-03	0.62
**Module T9**	CCNA2	1.3e-05	9.50	1.1e-03	1.70
	RAD51C	2.6e-04	6.00	4.7e-03	1.60
	DEPDC1B	8.0e-04	4.80	9.6e-03	1.50
	STMN1	9.3e-04	4.70	6.6e-04	1.80
	UBE2C	9.3e-04	4.70	8.4e-05	1.90
	UBE2S	9.3e-04	4.70	3.3e-04	1.80
	MCM3	9.3e-04	4.70	5.4e-03	1.60
	BIRC5	9.3e-04	4.70	7.8e-05	1.90
	STIL	9.3e-04	4.70	7.1e-03	1.60
	CENPM	9.3e-04	4.70	1.1e-03	1.70
	CHEK1	9.3e-04	4.70	4.6e-04	1.80
	CDK16	9.3e-04	4.70	5.1e-03	1.60
	ANGPT2	9.3e-04	4.70	9.7e-04	1.70
	MCM6	1.0e-03	4.70	1.9e-03	1.70
	TK1	1.2e-03	4.60	2.5e-04	1.80
	CDKN2C	1.2e-03	4.60	2.7e-03	1.70
	DLGAP5	1.2e-03	4.60	8.3e-03	1.60
	PLK4	1.2e-03	4.60	7.5e-04	1.80
	CDC20	1.5e-03	4.50	8.7e-03	1.60
	CDCA5	1.5e-03	4.50	2.6e-04	1.80
	MKI67	1.5e-03	4.40	1.6e-03	1.70
	FANCI	1.5e-03	4.40	7.8e-03	1.60
	KIF11	1.5e-03	4.40	5.0e-03	1.60
	TACC3	1.5e-03	4.40	8.1e-03	1.60
	CENPH	1.5e-03	4.40	2.8e-04	1.80
	MAD2L1	1.6e-03	4.40	5.2e-03	1.60
	PRKCA	2.2e-03	4.20	4.4e-03	1.60
	TTK	2.3e-03	4.20	2.1e-03	1.70
	KIF20A	2.3e-03	4.20	2.2e-03	1.70
	EFNA4	2.4e-03	4.20	3.2e-03	1.60
	EHMT2	2.4e-03	4.20	1.6e-03	1.70
	CDK1	5.1e-03	3.60	4.5e-03	1.60
	KIF2C	5.1e-03	3.60	6.6e-05	1.90
	SHCBP1	5.1e-03	3.60	5.7e-03	1.60
	NUP85	5.2e-03	3.60	3.7e-03	1.60
	CBX1	5.6e-03	3.50	4.7e-03	1.60
	CDK4	6.0e-03	3.50	3.1e-04	1.80
	WDR76	6.4e-03	3.50	1.1e-03	1.70

Table S2: Significant KEGG Pathways and GO Biological processes of premalignant modules.

Module	KEGG Pathways	GO Biological Processes
**N2**	Glycine, serine and threonine metabolism; Drug metabolism; Tryptophan metabolism; Complement and coagulation cascades; Cysteine and methionine metabolism; PPAR signaling pathway; Primary bile acid biosynthesis; Bile secretion; Steroid biosynthesis; Chemical carcinogenesis; Retinol metabolism; Metabolism of xenobiotics by cytochrome P450; Tyrosine metabolism; Arginine and proline metabolism; Cholesterol metabolism; Steroid hormone biosynthesis; One carbon pool by folate; Purine metabolism	cellular amino acid catabolic process; steroid metabolic process; fatty acid metabolic process; epoxygenase P450 pathway; cholesterol metabolic process; drug catabolic process; regulation of complement activation; purine ribonucleoside monophosphate catabolic process; regulation of immune effector process; regulation of humoral immune response; monocarboxylic acid metabolic process; dicarboxylic acid metabolic process
**N4**	Cell cycle; Oocyte meiosis; Progesterone-mediated oocyte maturation; Human T-cell leukemia virus 1 infection; p53 signaling pathway; Cellular senescence; DNA replication	mitotic spindle organization; microtubule cytoskeleton organization involved in mitosis; mitotic sister chromatid segregation; mitotic cytokinesis; spindle assembly checkpoint signaling; mitotic spindle checkpoint signaling; negative regulation of mitotic metaphase/anaphase transition; cytoskeleton-dependent cytokinesis; mitotic nuclear division; mitotic chromosome condensation; kinetochore organization; regulation of exit from mitosis; regulation of G2/M transition of mitotic cell cycle; cell cycle G2/M phase transition; regulation of cyclin-dependent protein serine/threonine kinase activity; anaphase-promoting complex-dependent catabolic process
**N5**	Protein processing in endoplasmic reticulum; Hepatitis C; Th17 cell differentiation; IL-17 signaling pathway; PPAR signaling pathway; Antigen processing and presentation; Cytokine-cytokine receptor interaction; Th1 and Th2 cell differentiation; Biosynthesis of unsaturated fatty acids; Bile secretion; Viral protein interaction with cytokine and cytokine receptor; Arginine biosynthesis; TNF signaling pathway; Cholesterol metabolism; B cell receptor signaling pathway; Epstein-Barr virus infection; MAPK signaling pathway; PD-L1 expression and PD-1 checkpoint pathway in cancer	cellular response to type I interferon; cytokine-mediated signaling pathway; defense response to virus; negative regulation of viral process; receptor-mediated endocytosis; regulation of viral genome replication; cellular response to interferon-gamma; regulation of ribonuclease activity; regulation of nuclease activity; interleukin-27-mediated signaling pathway; interferon-gamma-mediated signaling pathway; response to unfolded protein
**N7**	Phagosome; Complement and coagulation cascades; Fructose and mannose metabolism; HIF-1 signaling pathway; Viral protein interaction with cytokine and cytokine receptor; Human T-cell leukemia virus 1 infection; Cell adhesion molecules; Chemokine signaling pathway;; Natural killer cell mediated cytotoxicity; PPAR signaling pathway; Antigen processing and presentation; Leukocyte transendothelial migration; Galactose metabolism;	inflammatory response; neutrophil degranulation; neutrophil activation involved in immune response; neutrophil-mediated immunity; cytokine-mediated signaling pathway; regulation of T cell proliferation; regulation of immune response; microglial cell activation; chemokine-mediated signaling pathway; negative regulation of lymphocyte activation; positive regulation of MAPK cascade; cellular response to chemokine; dendritic cell differentiation; positive regulation of ERK1 and ERK2 cascade
**N10**	Complement and coagulation cascades; Bladder cancer	nuclear-transcribed mRNA catabolic process, nonsense-mediated decay; cellular protein metabolic process; SRP-dependent

		cotranslational protein targeting to membrane; regulation of lipid metabolic process; protein targeting to ER; regulation of apoptotic process; response to interferon-gamma; glutamate metabolic process; regulation of actin cytoskeleton organization; cytoplasmic translation; peptide biosynthetic process; regulation of ERK1 and ERK2 cascade
**N11**	Huntington disease; Prion disease; Ribosome; Oxidative phosphorylation; Non-alcoholic fatty liver disease; Alzheimer disease; Parkinson disease; RNA polymerase	mitochondrial translational elongation; mitochondrial translational termination; peptide biosynthetic process; mitochondrial translation; cellular macromolecule biosynthetic process; aerobic electron transport chain; mitochondrial ATP synthesis coupled electron transport; translation; cellular protein metabolic process; purine nucleotide metabolic process; lipoprotein catabolic process

Table S3: Survival analysis of genes from premalignant modules N3, N10, N7, and N5 on Chinese cohort tumour-adjacent normal samples. Hub genes with p-value ≤ 0.01 are shown.

	Gene	p-value	Hazard ratio
Module N3	PLK2	8.5e-07	2.50
	PLSCR1	8.2e-04	1.90
	ODC1	1.4e-03	1.80
	WWC1	1.6e-03	1.80
	RANGAP1	3.2e-03	1.70
	MYC	4.0e-03	1.70
	AVPR1A	4.1e-03	0.58
	DDX21	4.5e-03	1.70
	MCL1	5.3e-03	1.70
	BAG3	5.6e-03	1.70
	PHLDA1	6.1e-03	1.70
	PPRC1	6.5e-03	1.70
	B4GALT5	7.2e-03	1.70
	CDC37L1	7.3e-03	1.70
	SPRY2	8.1e-03	1.60
	RAB20	9.4e-03	1.60
Module N10	CDC42EP1	0.00086	1.90
	GGT1	0.00150	1.80
	TAGLN2	0.00180	1.80
	SDF2L1	0.00250	1.80
	DUSP5	0.00270	1.80
	GDF15	0.00360	1.70
	WDR13	0.00410	1.70
	TSPAN4	0.00450	0.59
	CKS2	0.00570	1.70
	IER3	0.00600	1.70
	ID3	0.00800	1.60
	PCNA	0.00830	1.60
	GNB2	0.00830	1.60
	CTSA	0.00850	1.60
	IFITM2	0.00980	1.60
Module N7	THBD	0.00035	2.00
	NUCB2	0.00045	1.90
	BCL2L1	0.00072	1.90
	MVP	0.00130	1.80
	PFKFB3	0.00210	1.80
	EPS8L3	0.00260	0.57
	CD59	0.00290	1.80
	GPX3	0.00290	1.70
	RBPMS	0.00310	1.70
	MYH4	0.00390	0.58
	ME1	0.00400	0.58
	ALDOA	0.00470	1.70
	BIRC3	0.00630	1.70
	NCKAP1L	0.00930	1.60
	ASCC3	0.00960	1.60
Module N5	GABBR2	0.00044	0.52
	JUN	0.00150	1.80
	SPINK1	0.00250	1.80
	AKAP12	0.00290	1.70
	TIPARP	0.00460	1.70
	FOS	0.00470	1.70
	EGR2	0.00580	1.70
	HSP90AA1	0.00580	1.70
	CPD	0.00760	1.60
	ERBB3	0.00900	0.61
	CPT1A	0.00940	1.60
	EPO	0.00950	0.62

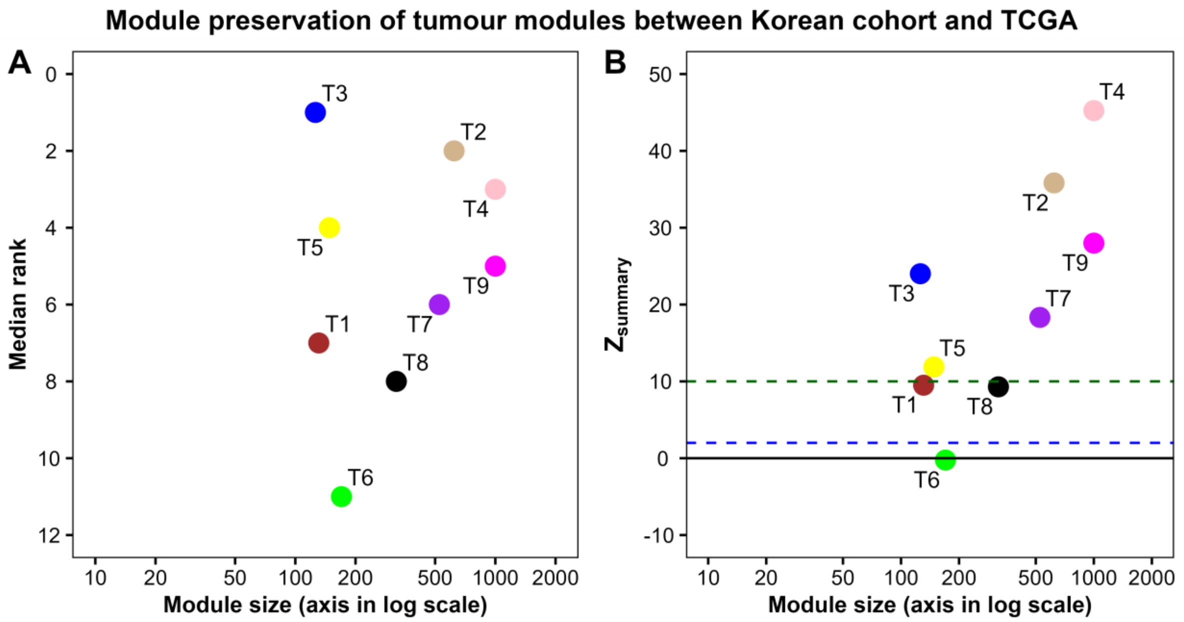

Figure S1: Module preservation statistics of Korean cohort tumour modules in TCGA tumour samples. Each point in the plots represents a module. (A) Median rank statistic as a function of module size. (B) $Z_{summary}$ statistic as a function of module size. The dashed blue and green lines correspond to thresholds Z =2 and Z=10, respectively.

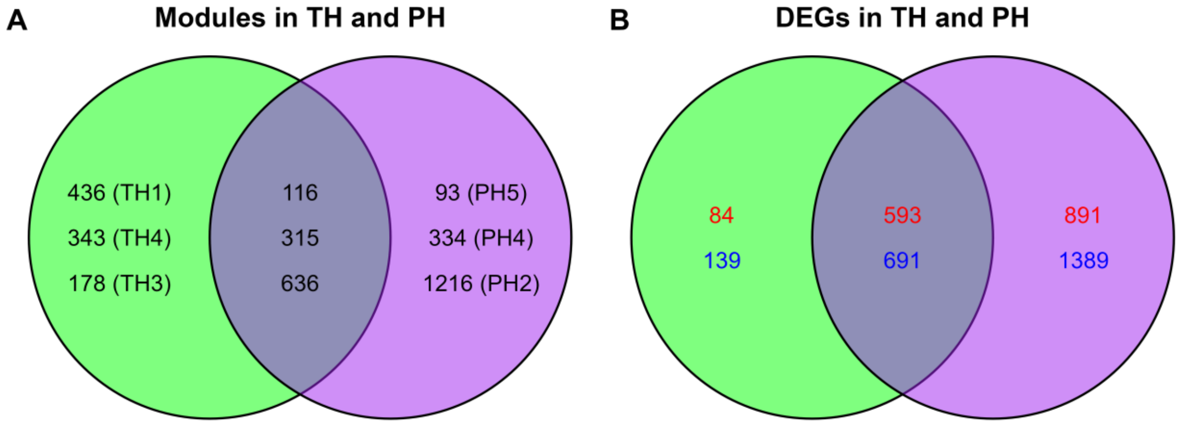

Figure S2: Comparison of precancerous to cancer progression in TH and PH treatment groups. (A) Venn diagram comparing precancerous-cancer modules in TH and PH. (B) Venn diagrams showing DEGs between cancer versus precancerous samples in TH and PH groups. Red and blue colour text indicates up and down regulated DEGs.

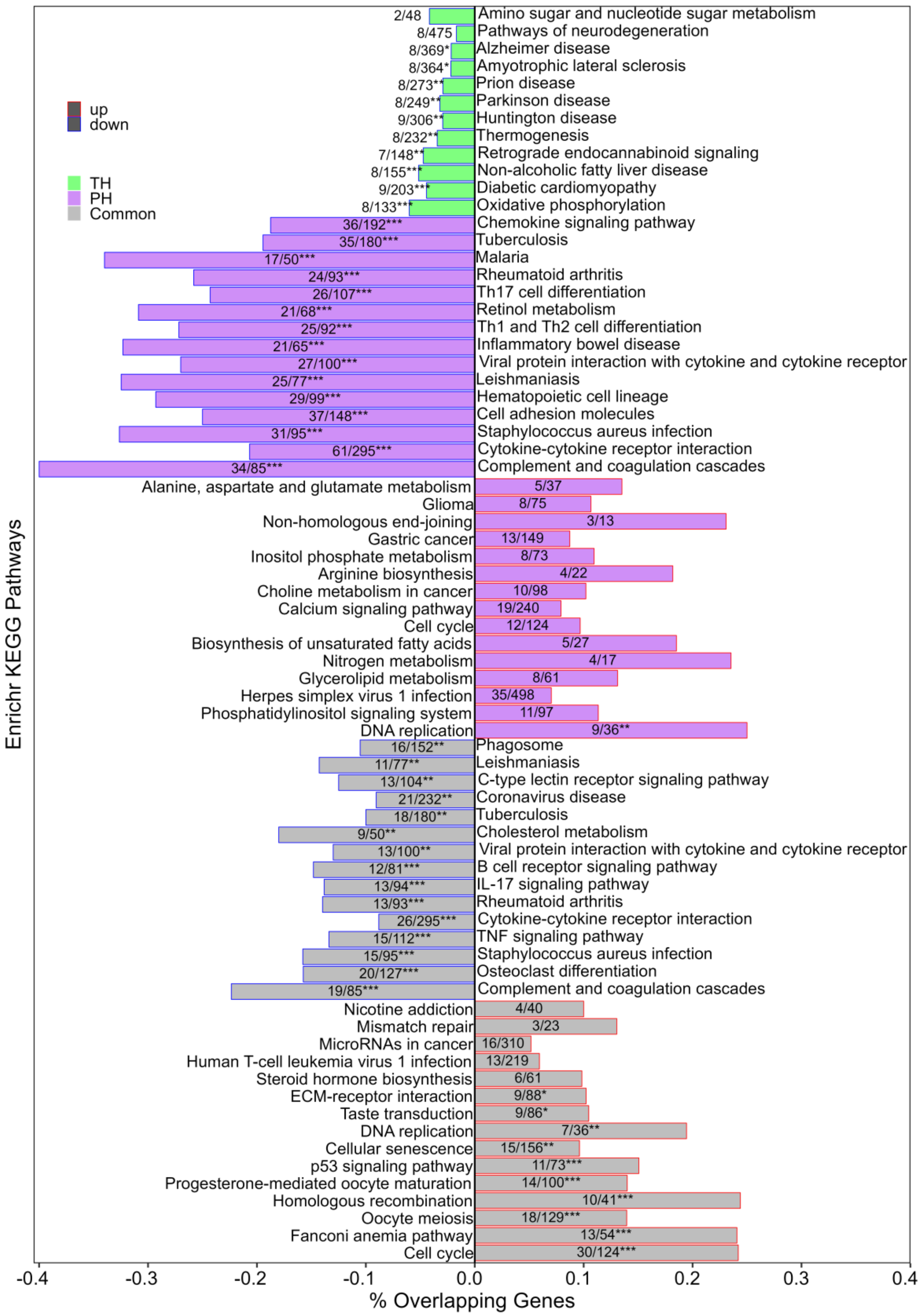

Figure S3: KEGG pathway enrichment of up and down regulated DEGs in different categories (TH only, PH only, Common to TH and PH). In each category, pathways with p-value < 0.05 within the top 15 pathways are shown. X-axis represents % of overlapping DEGs genes in each pathway. For down regulated pathways, negative value of % overlap is plotted. *** indicates adjusted p-value < 0.001, ** indicates 0.001 ≤ adjusted p-value < 0.01, * indicates 0.01 ≤ adjusted p-value < 0.05.

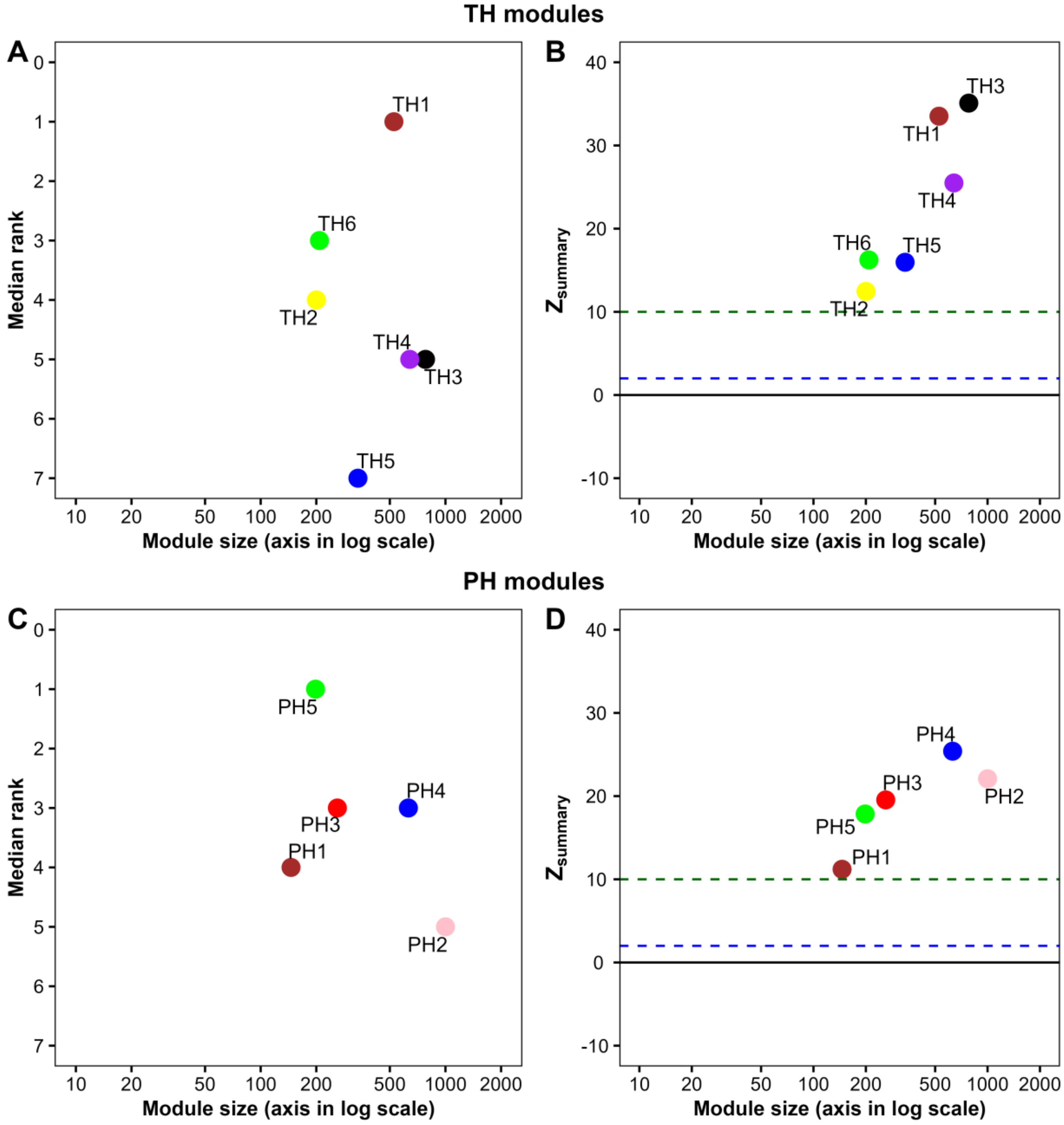

Figure S4: Module preservation statistics of Korean cohort precancerous-cancer modules in TCGA tumour and adjacent normal samples. Each point in the plot represents a module. (A)(C) Median rank statistic as a function of module size. (B)(D) Zsummary statistic as a function of module size. The dashed blue and green lines correspond to thresholds Z =2 and Z=10, respectively.

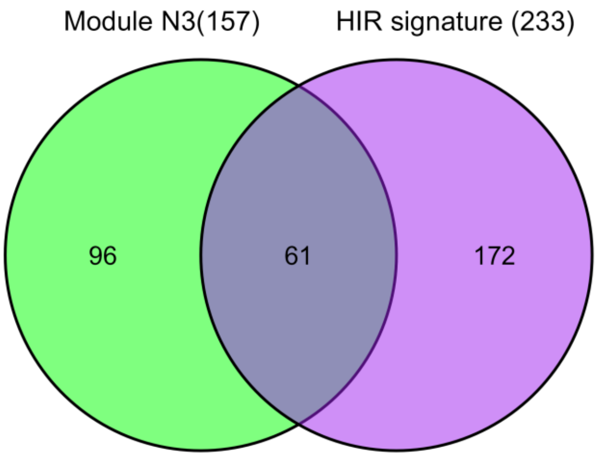

Figure S5: Venn diagram showing the intersection of module N3 genes and HIR signature.
